# Correction: MiR-HCC2 Up-regulates BAMBI and ELMO1 Expression to Facilitate the Proliferation and EMT of Hepatocellular Carcinoma Cells

**DOI:** 10.7150/jca.66366

**Published:** 2021-08-28

**Authors:** Jianying Yi, Yajie Fan, Le Zhang, Hong Wang, Ting Mu, Hong Xie, Huijie Gao, Min Liu, Shengping Li, Hua Tang

**Affiliations:** 1 Tianjin Life Science Research Center and Department of Pathogen Biology, Collaborative Innovation Center of Tianjin for Medical Epigenetics, School of Basic Medical Sciences, Tianjin Medical University, Tianjin 300070, China; 2 State Key Laboratory of Oncology in Southern China, Department of Hepatobiliary Oncology, Cancer Center, Sun Yat-sen University, 651 Dong-Feng Road East, Guangzhou 510060, China

We recently have noticed three inadvertent mistakes due to our carelessness and high similar images in the preparation of the figures and would like to correct them.

(1) The migration image of pcDNA3 group for Huh7 cells in Figure 2A was misplaced. We had inserted the image of ASO-NC group for Huh7 cells in the same panel by mistake. We have put the right picture to show the migration image for pcDNA3 group in Figure 2A.

(2) Due to our carelessness in the generation of the figure, the invasion images of pSilencer-NC group and shR-ELMO1 group for Huh7 cells in Figure 5D respectively represented an erroneous duplication of the image of pcD3-Flag/KBE group for Huh7 cells in Figure 5C and the image of ASO-NC group for Huh7 cells in Figure 2A. We have put the right pictures to show the invasion images for pSilencer-NC group and shR-ELMO1 group of Huh7 cells in Figure 5D.

(3) The invasion images of pcDNA3+pSilencer-NC group and pri-miR-HCC2+pSilencer-NC group for Hep3B cells in Figure 6F were misplaced. The images of pri-miR-HCC2+pSilencer-NC group for Huh7 cells in Figure 6F and pSilencer-NC group for Huh7 cells in Figure S3A were duplicated respectively here due to our carelessness in the generation of the figure. We have put the right pictures to show the invasion images for pcDNA3+pSilencer-NC group and pri-miR-HCC2+pSilencer-NC group of Hep3B cells in Figure 6F.

The correction does not change the overall conclusions of this paper. All of the data have been checked carefully, and no errors exist in the corrected version.

## Updated Figure 2, Figure 5 and Figure 6

## Figures and Tables

**Figure 2:**
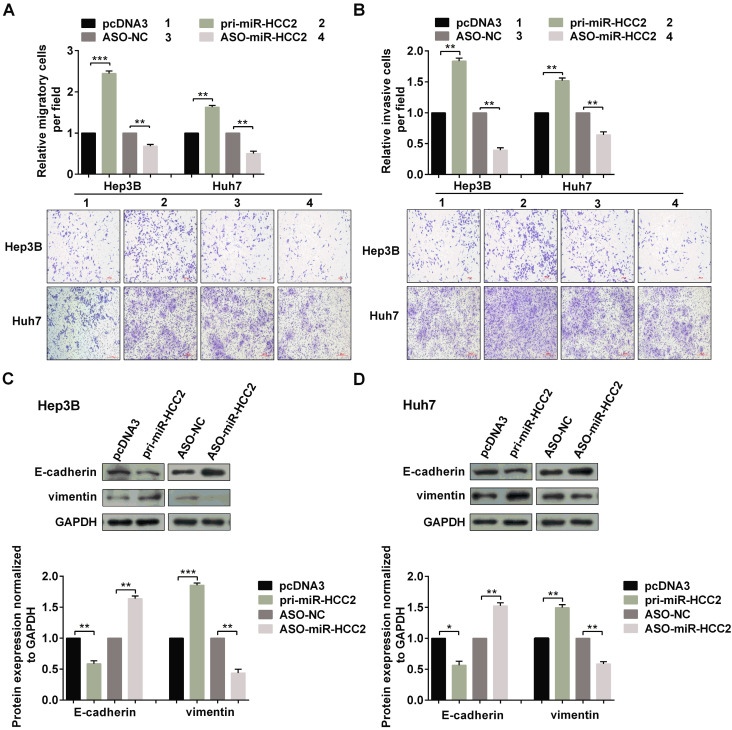
The corrected new figure is shown.

**Figure 5:**
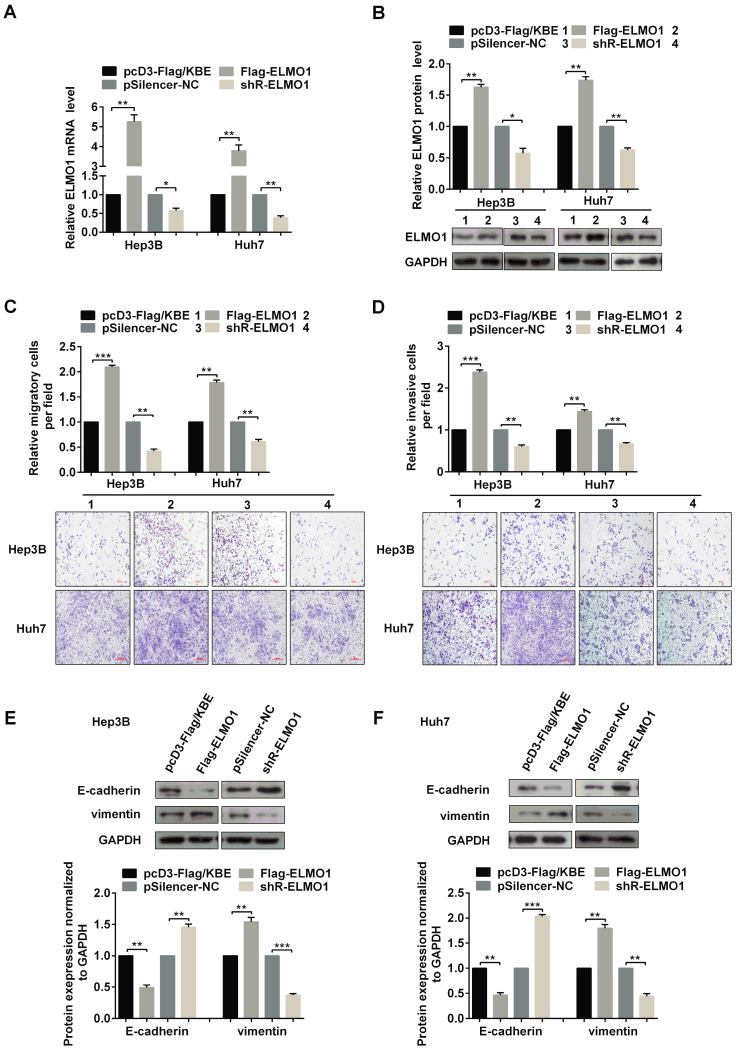
The corrected new figure is shown.

**Figure 6:**
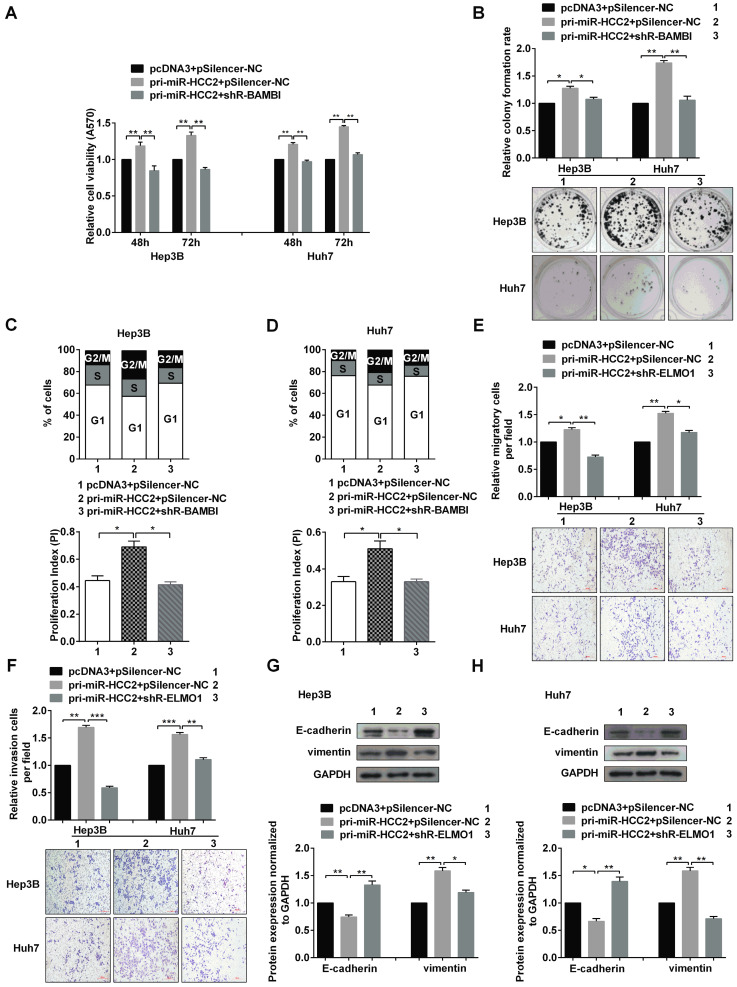
The corrected new figure is shown.

